# Reporters of Cancer Stem Cells as a Tool for Drug Discovery

**DOI:** 10.3389/fonc.2021.669250

**Published:** 2021-04-22

**Authors:** Amrutha Mohan, Reshma Raj R., Gayathri Mohan, Padmaja K. P., Tessy Thomas Maliekal

**Affiliations:** ^1^ Cancer Research, Rajiv Gandhi Centre for Biotechnology, Thiruvananthapuram, India; ^2^ Centre for Doctoral Studies, Manipal Academy of Higher Education, Manipal, India

**Keywords:** cancer stem cells, drug resistant CSCs, metastasis initiating cells, fluorescent reporters, drug screening and discovery

## Abstract

In view of the importance of cancer stem cells (CSCs) in chemoresistance, metastasis and recurrence, the biology of CSCs were explored in detail. Based on that, several modalities were proposed to target them. In spite of the several clinical trials, a successful CSC-targeting drug is yet to be identified. The number of molecules screened and entered for clinical trial for CSC-targeting is comparatively low, compared to other drugs. The bottle neck is the lack of a high-throughput adaptable screening strategy for CSCs. This review is aimed to identify suitable reporters for CSCs that can be used to identify the heterogeneous CSC populations, including quiescent CSCs, proliferative CSCs, drug resistant CSCs and metastatic CSCs. Analysis of the tumor microenvironment regulating CSCs revealed that the factors in CSC-niche activates effector molecules that function as CSC markers, including pluripotency markers, CD133, ABCG2 and ALDH1A1. Among these factors OCT4, SOX2, NANOG, ABCG2 and ALDH1A1 are ideal for making reporters for CSCs. The pluripotency molecules, like OCT4, SOX2 and NANOG, regulate self-renewal, chemoresistance and metastasis. ABCG2 is a known regulator of drug resistance while ALDH1A1 modulates self-renewal, chemoresistance and metastasis. Considering the heterogeneity of CSCs, including a quiescent population and a proliferative population with metastatic ability, we propose the use of a combination of reporters. A dual reporter consisting of a pluripotency marker and a marker like ALDH1A1 will be useful in screening drugs that target CSCs.

## Introduction

Despite the advancement in drug development, chemoresistance and metastasis are the primary reasons for high cancer mortality. Recent research has identified that cancer stem cells (CSCs), a rare sub-population within cancer cells possessing self-renewal ability, drug resistance and high metastatic ability, are the reason for the relapse of the disease. Even though CSCs were identified in 1994 in AML (1), their relevance in solid cancer was widely explored after the identification of CD133^+^ population in colorectal cancer in 2007 (2, 3). Research in this field for more than a decade has reinforced the importance of this self-renewing population in cancer progression, metastasis, chemo-resistance and recurrence. The classical chemotherapeutic agents induce apoptosis by damaging DNA and/or by inhibiting mitotic division. As this therapeutic option is valid only for highly dividing cancer cells, slow dividing CSCs escape the drugs, leading to relapse. In addition, CSCs get a survival advantage by the high expression of the ATP-binding cassette (ABC) transporters, aldehyde dehydrogenases (ALDHs), and antiapoptotic molecules (4), acquiring drug resistance. Metastasis is a dynamic multistep process including the escape from the primary tumor, intravasation to the systemic circulation, extravasation at metastatic site, organ-seeding, and final metastatic colonization with the tumor outgrowth. Importantly, only a minority of the dispersed tumor cells expressing the stemness markers characteristic of CSCs, known as metastasis initiating cells (MICs), survives all these steps to initiate metastasis at a distant site. Given the importance of CSCs in bad prognosis, strategies for targeting them is gaining importance in cancer drug discovery (5–13).

## Clinical Trials for Targeting CSCs

The clinical relevance of CSCs is largely attributed to their resistance to chemotherapy and radiotherapy, which is mainly due to the quiescent nature of CSCs. The treatment-induced and microenvironment-induced de-differentiation of cancer cells to CSCs also poses a challenge in tumor eradication. CSC properties, including self-renewal ability, therapy resistance and metastasis, primarily depend on the aberrant expression of CSC-specific molecules and their interaction with CSC-niche. Thus, the current approaches for eradicating this population include differentiation of CSCs, targeting drug efflux molecules and other surface markers of CSCs, and inhibition of the signaling pathways sustaining CSCs (5). Various studies have been conducted to disrupt the CSC niche using inhibitors of CXCR4 or FAK (**Table 1**). Inhibitors of drug resistant molecules, MDR proteins including ABCG2 have been targeted to abolish drug resistant CSCs (**Table 1**). As reviewed recently, a majority of the attempts in clinical trials are for targeting the important signaling pathways like Notch, WNT, Hedgehog and Hippo along with immunotherapeutic approaches, both in solid cancers and hematological malignancies (12, 29)

**Table 1:** The drugs targeting CSCs in clinical trials.

Signaling Pathway	Drug	Effective in preclinical studies		Clinical trials	
		Cancer	Reference	NCT no. (cancer)	Outcome
**Notch**	MK-0752	Breast cancer	(14)	NCT00645333 (Breast cancer)	Not reported
	PF-03084014	Triple negative breast cancer	(15)	NCT01981551 (Desmoid tumors)	PR in 29%; SD in 29%
	Demcizumab	Non-squamous non-small cell lung cancer (NSCLC)	(16)	NCT02289898 (locally advanced solid tumors)	Placebo vs demcizumab ORR: 44.2% vs 33.1% CBR: 70.6% vs 74.3% Median PFS: 5.5 months in both arms
**WNT**	PRI-724	Colorectal cancer	(17)	NCT01764477(pancreatic cancer)	SD in 40.0%; Median PFS: 2 months (range 0.7-7.7)
	DKN-01	Nil	NA	NCT02013154 (solid tumors)	Encouraging early efficacy signals
**Hedgehog**	Glasdegib	Acute myeloid leukemia	(18)	NCT01546038 (acute myeloid leukemia)	CRs in 17.0% Median OS: 8.8 months
	Vismodegib	Breast cancer	(19)	NCT00833417 (basal cell carcinoma)	ORR 48.5% in metastatic BCC group and 60.3% in the locally advanced BCC group; Median DoR: 14.8 months and 26.2 months, respectively.
**Hippo**	Pivonedistat	Chronic myeloid leukemia	(20)	NCT01862328 (solid tumors)	CR in 3.7%; PR in 18.5%; SD in 78.6% treated at MTD; Median DoR: 5.9 months
**JAK**	Ruxolitinib	Gastric cancer	(21)	NCT01594216 (breast cancer)	Not reported
**PI3K**	BYL719	Medulloblastoma	(22)	NCT01613950 (gastric cancer)	Not reported
**EGFR**	Bevacizumab	Melanoma	(23)	NCT01190345 (breast cancer)	Not reported
**CXCR4**	BL-8040 Plerixafor	Nil Colon cancer	NA (24)	NCT02907099 (solid tumors) NCT00512252 (acute myeloid leukemia)	Not reported ORR 46%; median DoR 19.8 months. The median OS was 8.2 months with RFS of 9.0 months. One-year OS and RFS were 37% and 42.9%.
**FAK**	Defactinib/VS-6063	Breast cancer	(25)	NCT04439331 (solid tumors)	Not reported
**BCL2**	Venetoclax	Acute myeloid leukemia	(26)	NCT03466294 (acute myeloid leukemia)	Not reported
**MDR**	Dofequidar/MS-209	Breast cancer	(27)	NCT00004886 (solid tumors)	Not reported
**ABCG2**	Cyclosporin			NCT00983424 (breast cancer)	Not reported
**EpCAM**	Catumaxomab	Pancreatic carcinoma	(28)	NCT00836654 (malignant ascites)	Increased OS

BCC, basal cell carcinoma; CBR, clinical benefit rate; CR, complete response; DLL, Delta-like ligand; DoR, duration of response; MTD, maximum tolerated dose; ORR, objective response rate; OS, overall survival; PFS, progression-free survival; PR, partial response; PFA, prostate-specific antigen; SD, stable disease.

A comprehensive analysis of the reported clinical trials in comparison to their preclinical analysis showed that the majority of the drugs that were tried in trials were already in use for other diseases or for cancer itself, but not specifically for CSCs (**Table 1** **)**. The CSC-targeting efficacy of Hedgehog inhibitor Vismodegib is established in preclinical studies of breast cancer (30). The Hedgehog inhibitors Vismodegib and Sonidegib are FDA approved drugs for basal cell carcinoma, and their clinical trials have shown some acceptable response, though CSC specific activities were not evaluated in those trials (31, 32). When these drugs were combined with conventional chemotherapeutic agents, there was no advantage observed for Hedgehog inhibitors (12, 33). The clinical trials for inhibitors of WNT pathway and Notch pathway have shown some positive aspects in early phase trials, but their therapeutic potential and efficacy in targeting CSCs are yet to be proved (12, 33). Thus, most of the inhibitors for the well-established self-renewal pathways, which influence many cellular functions are not promising therapeutic agents in clinics, as summarized in **Table 1**. The clinical trials for targeting some other pathways, cell surface molecules, MDR proteins, niche interactions, etc. are ongoing. Since CXCR4-mediated interaction of CSCs to “CSC niche” is critical for their maintenance, targeting this molecule using its antagonist Plerixafor has shown a very promising result in acute myeloid leukemia with 46% of complete remission (34). The phase II/III trial of targeting the CSC marker, EpCAM using a monoclonal antibody Catumaxomab in ascites secondary to epithelial cancer has demonstrated a clear clinical benefit in patients, leading to its approval in Europe (35). Currently, it is being used for immunotherapy using CAR-T cells recognizing EpCAM for advanced solid tumors, the results of which are awaited. To conclude, several approaches are adopted to target CSCs, which have shown some promise but their therapeutic efficacy in larger clinical trials have to be proved. Irrespective of the strategy used, the efficiency of the drug is evaluated in clinical as a well as preclinical analysis using certain assays. Currently, CSCs are evaluated by FACS-based detection of the surface markers, sphere formation assay, soft agar colony formation assays, *in vitro* and *in vivo* serial passaging as well as *in vivo* limiting-dilution tumorigenicity assays using immunocompromised mice. Hence, ultimately, the screening assays define the efficiency of the drug. If the screening assay is inefficient in representing all the heterogeneous CSCs, it will lead to the failure of the drug in clinical trials.

In a classical drug discovery program, the majority of the drugs that enter the screening program fails in the “Valley of Death”, and a very few candidates emerge as drugs for clinical use. Since the *in vitro* assays used to ascertain CSC properties are not compatible with high-throughput screening platforms, it is very difficult to have a screening strategy for CSC-targeting drugs. Hence, the evolution of clinical trials for CSC targeting is by exploiting “drug repositioning”, which can bypass the problem of bioavailability and safety problems (13, 36). Yet, many of the drugs that entered clinical trials did not improve therapeutic efficacy (37). Hence, there is an increasing demand for new drugs for CSC-targeted therapy. In this context, we propose that CSC-targeting drug screening platforms can be developed using reporters of CSCs, which have been recently published (38–45). These fluorescent reporters can be easily adapted for large scale screening. Considering the heterogeneity of CSCs, including a quiescent population and a proliferative population with the metastatic ability (46), we propose the use of a combination of reporters. A dual reporter for a pluripotency marker and a marker like ALDH1A1 or ABCG2 will be useful in screening drugs that target CSCs. These dual reporters will ensure the representation of all the heterogeneous CSCs in the screening assay.

## The Factors Contributing to CSC Properties

**Figure 1:**
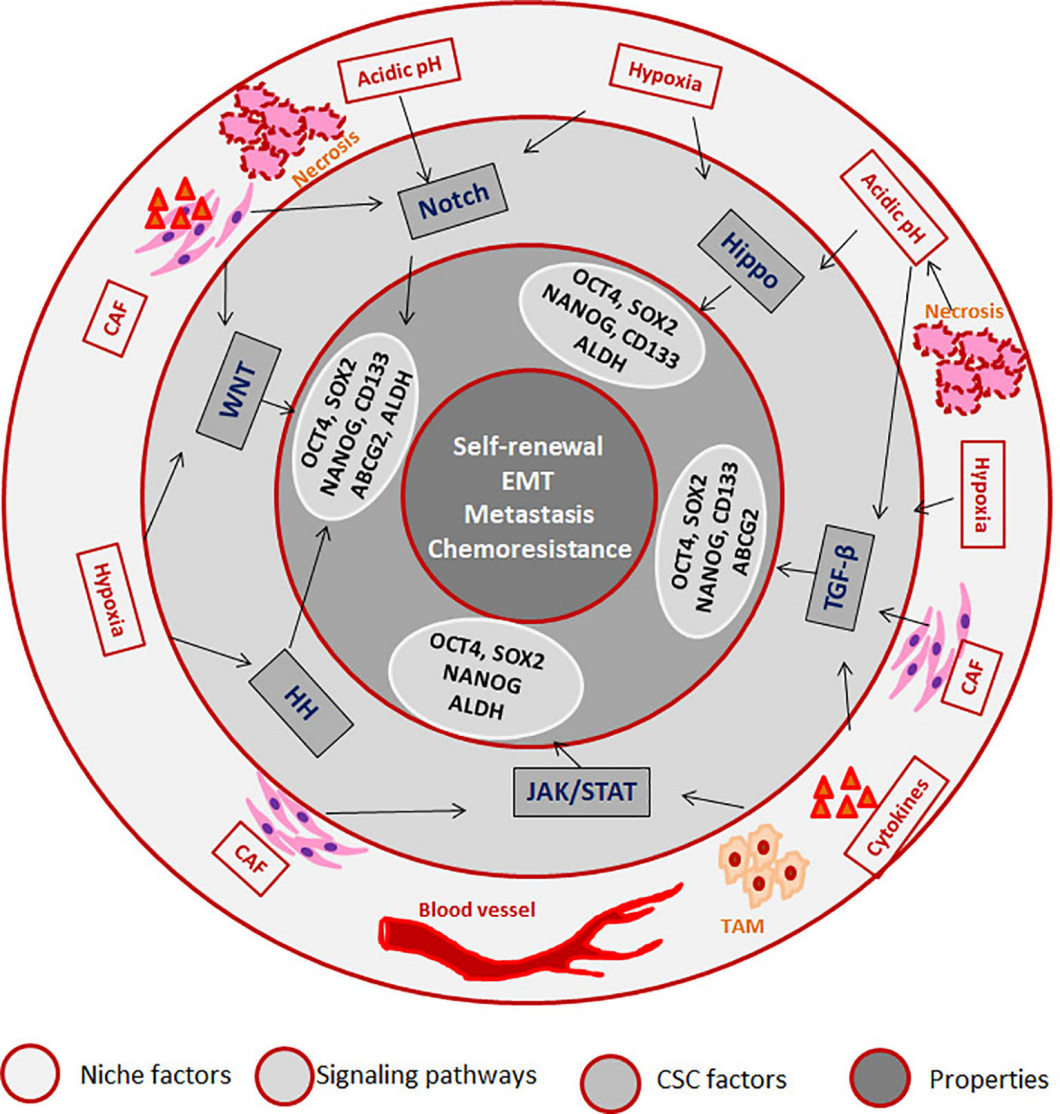
The role of “CSC niche” in regulating cancer stem cell properties. The Tumor associated macrophages (TAM) secretes cytokines initiating JAK/STAT and TGF-β signaling pathways. The regions where blood supply reduces creates hypoxia, which activates Hedge Hog (HH) WNT, Notch and Hippo signaling pathways. The regions where there is no oxygen and nutrient supply undergo necrosis, which creates an acidic pH that activates Notch and Hippo. Cancer associated fibroblasts (CAFs) also secret factors that initiate different signaling pathways. As a result of the pathway activation, several CSC-associated molecules are activated that results in the induction of CSCs.

CSCs can be defined as a subpopulation that exhibits self-renewal property, while there are accumulating evidences to show that different pools of CSCs co-exist in a single tumor that can have varied properties including quiescence, drug resistance, epithelial to mesenchymal transition (EMT), invasion and metastasis (47). But, it is not necessary that all CSCs possess all the above characteristics. Recent reports underscore the induction of stemness in cancer cells according to the microenvironment in which the cancer cell resides. According to this induction theory, CSC is a state with high plasticity where all the CSC-associated properties are regulated by the “CSC niche”, which in turn can be attributed to the cytokine levels, hypoxia, acidic pH, cancer associated fibroblasts (CAFs) and the signaling pathways initiated thereby (33). Hypoxia, the low oxygen concentration in the tumor microenvironment, can influence the tumor cells, infiltrating blood cells and CAFs to alter the secreted cytokines and growth factors in a HIF-1α-dependent manner, which results in the acquisition of CSC properties (48). HIF-1α can impart drug resistance through transcriptional up-regulation of MDR1, ABCG2 and MRP2, while it induces stemness by regulating CD133 and Notch pathway (48). Hypoxia regulates epithelial to mesenchymal transition (EMT) which in turn, up-regulates EMT/β-catenin/STT3 axis as well as PD-L1, thereby helping in the immune evasion of CSCs (49). Hypoxia and other secreted factors collectively up-regulate certain oncogenic stemness molecules like Notch1, which is reported as a marker for HNSCC stem cells (50). The role of hypoxia in hematological malignancies is also studied in detail, and the important findings are reviewed elsewhere (51). In multiple myeloma cells, hypoxia activates EMT program and increases the expression of stem cell transcription factors, ABCG2 and ALDH1 to impart cancer stem cell phenotype and metastasis (52, 53). Hypoxia is known to impart drug resistance through the up-regulation of P-gp and FAK (54, 55). The developmental pathway up-regulated in CSCs are WNT, Hedgehog and Hippo, and the biological activities of CSCs are regulated by several pluripotent transcription factors, such as OCT4, SOX2, NANOG, KLF4, and MYC (5, 12). We have summarized how the factors and signaling pathways existing in “CSC niche” regulate properties associated with CSCs in **Figure 1**. The role of these pathways in the regulation of CSCs is extensively reviewed recently (5, 12).

The “CSC niche” provides an immunosuppressive environment for the survival and growth of CSCs, which will enable tumor growth, chemoresistance and metastasis. In the primary site, the tumor microenvironment is adapted to induce stemness, acquire chemoresistance and induce metastatic ability for CSCs to convert them to MICs. The “CSC niche” in the metastatic site is also important in metastatic spread of the disease. Accumulating evidences suggest the existence of pre-metastatic niches that are permissive to the colonization of specific CSCs (56). These niches allow the CSCs to remain dormant during chemo/radiotherapy, and leads to recurrence after the withdrawal of the therapeutic agent. Bone marrow microenvironment is an important CSC-niche for both hematological malignancies as well as solid tumors. The leukemia stem cells are shown to hijack the hematopoietic stem cell niche to acquire and maintain CSC properties, including self-renewal, drug resistance and metastasis (57, 58). Bone marrow is also serving as survival place for breast cancer cells, where they metastasize to remain dormant till a favorable condition is regained (59–62).

Currently, based on the available literature, it can be summarized that the cancer cells acquire self-renewal property and become CSCs, depending on the signaling network existing in the “CSC niche”. They can further acquire drug resistance and metastatic ability in response to the tumor microenvironment, generating CSCs, exhibiting drug resistance and/or metastasis. This drug resistant CSC or MIC will be responsible for the bad prognosis, and thus should be targeted. Such reporters identifying metastatic CSCs or drug resistant CSCs should be used for screening drugs in preclinical models. The following sections will briefly examine the pathways and the consistent intermediate molecules and markers that regulate drug resistance and metastasis.

## CSCs and Drug Resistance

**Figure 2:**
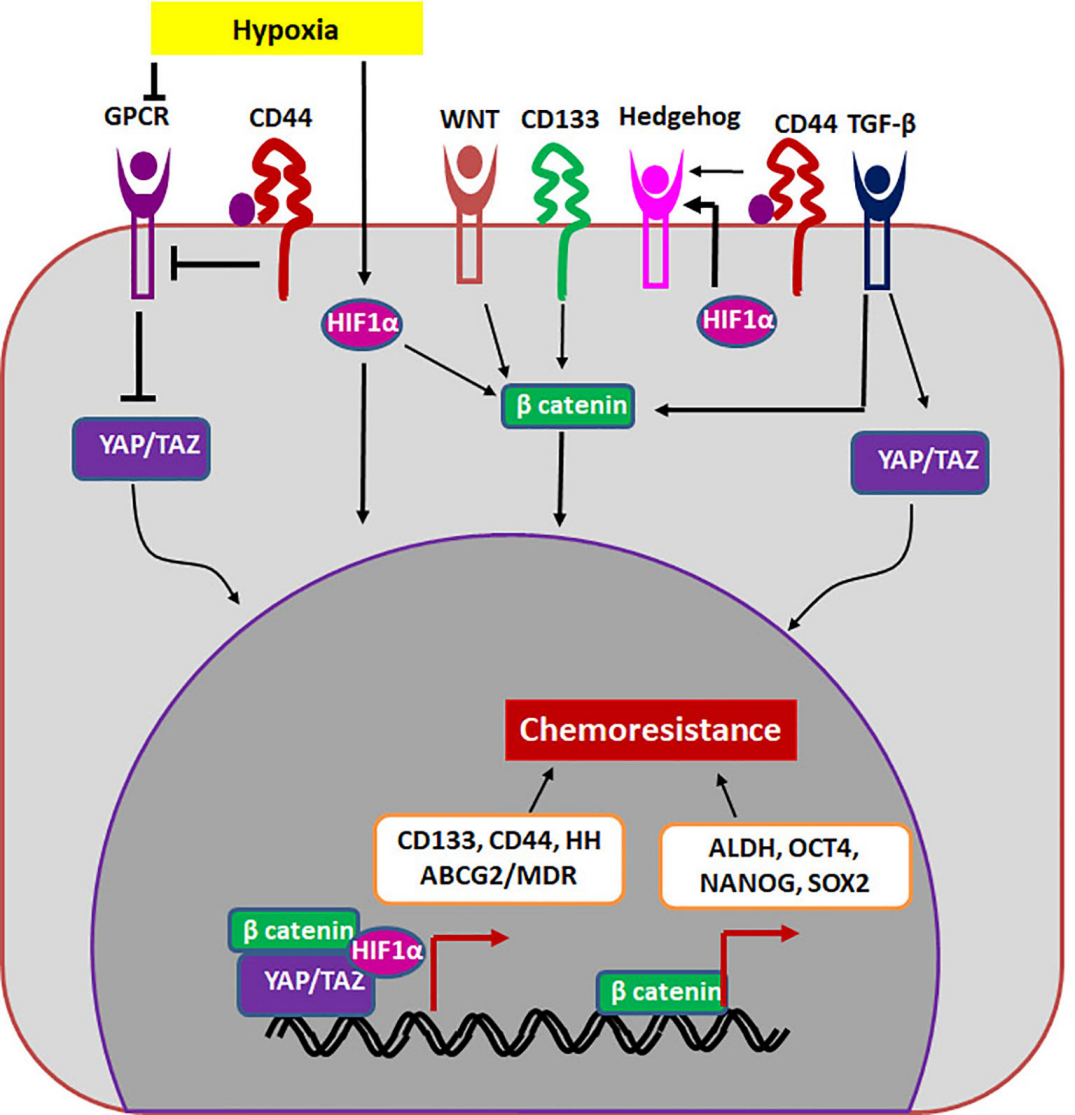
The regulation of drug resistant CSCs. The niche factors activates several self-renewal pathways like HIF1α, WNT, Hedgehog or TGF-β that regulate chemoresistance in a β-catenin dependent way. Other self-renewal markers like CD44 and CD133 can also regulate chemoresistance. CD133 regulates β-catenin, while CD44 regulates Hippo signaling (YAP/TAZ) to induce chemoresistance. The transcription complexes indicated in the figure results in the up-regulation of molecules like ALDH and MDR proteins including ABCG2.

If we extrapolate the classical stem cell properties to cancer, a CSC should be a cell that is G_0_-arrested, and divides asymmetrically to give rise to a non-dividing cell and a dividing differentiating cell. The G_0_-arrested, low cycling CSC population in cancer will not be responsive to classical chemotherapeutic agents that target dividing cells. These cells that overcome therapy can re-establish the tumor since they possess the tumor initiating property, showing the role of CSCs in drug resistance and relapse. In another scenario, cancer cells up-regulate drug resistant molecules in response to therapy to overcome the effect of the drug. Though a sub-population of it can acquire self-renewal ability and become CSCs, all these drug resistant population may not have the self-renewal ability to re-create the entire tumor to cause recurrence. Therefore, it is important to segregate general drug resistant population from the drug resistant CSCs. Here, we postulate that molecules and pathways that regulate both self-renewal and drug resistance are important in sustaining the drug resistant CSCs. Even though many CSC markers are shown to regulate chemoresistance, there are two molecules, ABCG2 and ALDH that directly regulate drug resistance. The other self-renewal molecules regulate drug resistance by up-regulating either multidrug resistant proteins or ALDH (**Figure 2**).

Over-expression of drug efflux molecules like ABC transporter molecules is one of the strategies adopted by cancer cells to overcome chemotherapy. Although all the different ABC transporters can do drug efflux, ABCG2 is usually associated with embryonic stem cells, adult stem cells and CSCs, but not exclusive to them (63). These drug efflux molecules transport substrates across the membrane to reduce their intracellular concentration, where the substrates can vary from xenobiotics to a variety of chemotherapeutics (63). ALDH is an enzyme catalyzing the conversion of cellular aldehydes to their corresponding carboxylic acids, which helps in detoxifying the chemotherapeutic agents by oxidizing the aldehyde groups of the drug. Though all ALDH forms can do this enzymatic detoxification, the self-renewal related isoforms like ALDH1A1 can regulate drug resistance by the up-regulation of drug efflux molecules through retinoic acid (RA) signaling. Thus it is evident that ABCG2 and ALDH1A1 are useful in identifying drug resistant CSCs.

### CSCs and Metastasis

In continuation to the report from Weinberg’s lab (64), several reports reinforced the importance of EMT, a prerequisite for metastasis, in the acquisition of CSC characteristics. At a closer look, there is a considerable overlap in the signaling pathways and the markers for CSCs and metastatic cells. Also, the similarity in the hierarchical organization of metastatic lesions and primary tumor led to the assumption that CSCs are the metastasis initiating cells (MICs). But recent reports show that there are distinct populations with tumor initiating capacity or metastasis initiating capacity (65). Among breast cancer circulating tumor cells, CD44^+^CD24^+^ALDH1^+^ subpopulation show an increased tumorigenic potential, while EpCAM^+^CD44^+^CD47^+^MET^+^ subset denotes cells with high metastatic ability (66). In a *MMTV*-*PyMT* mammary tumor model, Lin^−^CD90^−^ALDH^Hi^ cells mark the tumor-initiating population while Lin^−^CD90^+^CD24^+^ cells are the high metastatic cells (65). It was also noted that a subset of Lin^−^ALDH^low^ cells can replenish the Lin^−^ALDH^Hi^ fraction (65).

**Figure 3:**
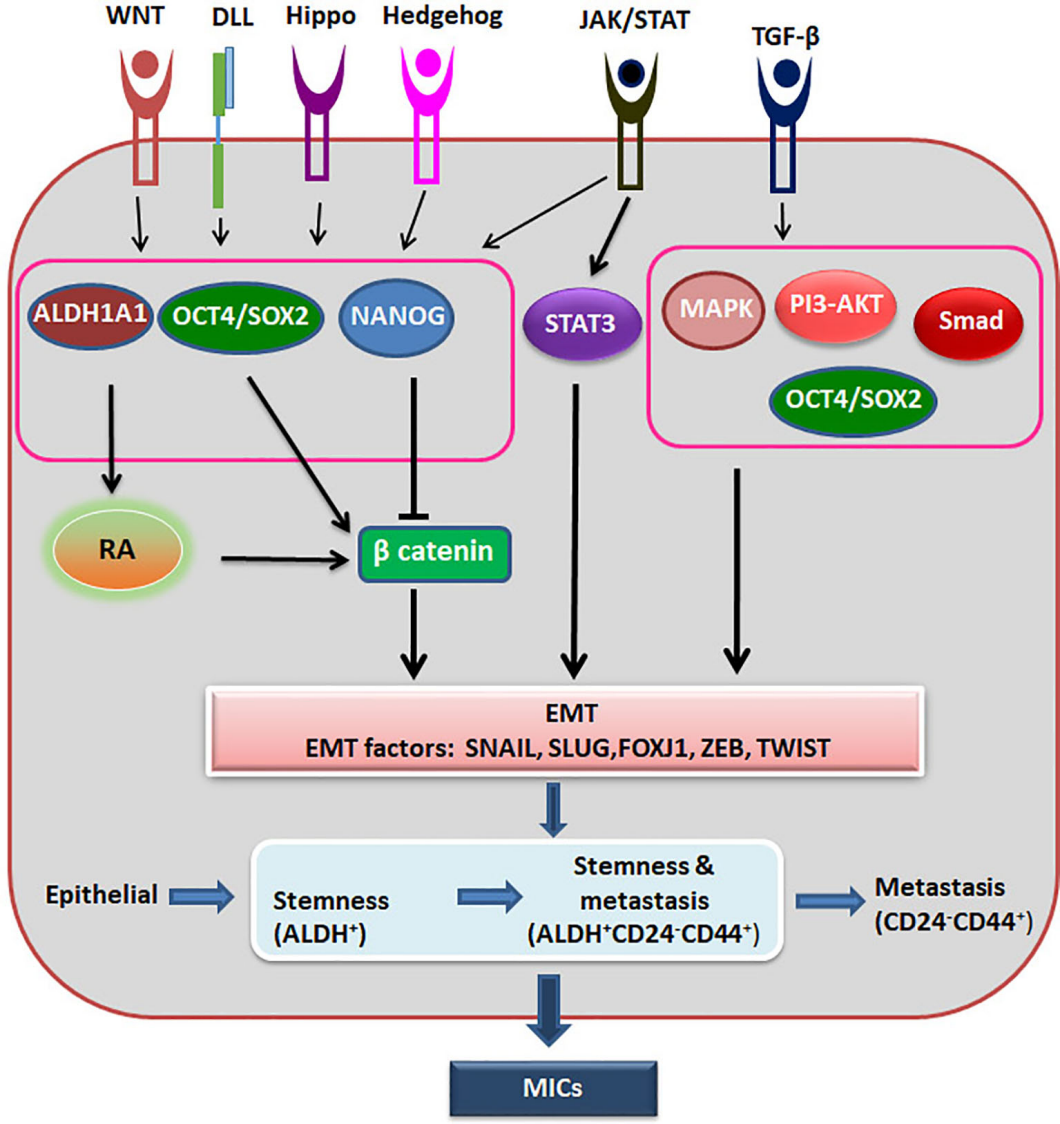
The regulation of Metastatic CSCs. Different signaling pathways activate ALDH1A1, OCT4 and SOX2, which activates β-catenin to induce EMT. At the same time OCT4/SOX2 regulates EMT in a β-catenin independent way also. NANOG inhibits β-catenin, but activates EMT through other pathway. When there is induction of EMT, epithelial cells might acquire stemness and metastatic ability, and gradually lose the stemness. The hybrid cells showing stemness property and metastatic property together are the metastasis initiating cells (MICs).

Recent advancement in the field suggests that epithelial-mesenchymal plasticity (EMP) is the driving force of CSCs during metastasis, as reviewed recently (67). They argue that EMT has three stages, where cells can have more epithelial nature or a hybrid epithelial and mesenchymal nature or extreme mesenchymal characteristics. They have summarized the evidences to show that the cells with the hybrid nature are the actual metastasis initiating cells possessing CSC characteristics, while the extreme mesenchymal cells are devoid of tumor initiating properties (67). It is also postulated that the stemness of circulating tumor cells resides in a window where it shows the hybrid characteristics and express markers like ALDH (**Figure 3**). With the progression of metastasis, these ALDH^Hi^ cells can acquire markers like CD24^-^CD44+ with progressive loss of ALDH. On the metastatic site, this loss of expression can be reversed based on the niche to start colonization (68).

### Identification of CSCs

Given the importance of CSCs in tumor progression and prognosis, several attempts were made to identify and characterize CSCs based on the markers they express, either cell surface markers or other functional markers like pluripotency markers (OCT4, SOX2 and NANOG) or high ALDH activity. The CSC markers generally used to detect different forms of malignancy are summarized in **Table 2**. There are several markers that can identify CSCs with drug resistance and metastatic potential, either alone or in combination with other markers (**Table 3**). The primary mode of detection of CSCs is FACS using relevant antibodies, involving several steps that make it difficult to use it for screening drugs. The limiting factors are the cost of the antibody and the steps involved in the process. So, there are some alternative approaches, like reporter constructs of CSC markers, to detect CSCs.

**Table 2:** Markers of CSCs for different malignancies.

Types of Cancer	Marker Signature of CSCs
**Hematological malignancy**
Acute lymphoid leukemia	CD34+/CD38−/CD19+ (69)
Acute myeloid leukemia	CD34+/CD38− (70)
	NANOG (71)
	OCT4 (72)
	ALDHA1 (73)
Chronic myeloid leukemia	ALDHA1 (73)
	CD34+/CD38−/CD26+ (74)
	ALDHA1/SOX2/NANOG (32)
Hodgkin lymphoma	CD27+/ALDH+/(CD19+/CD20+) (75)
**Solid tumors**	
Breast cancer	OCT4/NANOG/(CD)44+/CD20 (76)
	LGR5 (77)
	CD44/OCT4/NANOG/SOX2 (29)
	ALDH1A1 (78)
Colorectal cancer	OCT4/NANOG (79)
	AGR2/LGR5 (80)
	CD133+ (2)
	CD133/CD44 (81)
Glioblastoma	CD133+ (82)
Hepatocellular cancer	CD133+/EpCAM+ (83)
	CD90+/CD44 (84)
Lung cancer	CD133+ (85)
	CD117+ (86)
	ALDH+ (87)
	OCT4A/CD133/ALDH (88)
	CD44+ (89)
	SOX2 (90)
Medulloblastoma, melanoma	CD133+ (91)
Ovarian cancer	CD44/CD105/CD106 (92)
	CD133+ (93)
	ALDH+/CD133+ (94)
	CD44+/CD117+ (95)
Pancreatic cancer	CD44+/CD24+ (96) CD133+/ALDHA1 (97)
	CD44/OCT4/NANOG/SOX2 (98)
Prostate cancer	CD133/CD44/OCT4/NANOG/SOX2/ABCB1/ABCG2/ABCC1 (99)

**Table 3:** Markers of heterogenic CSCs.

Marker	Tumour Initiating Capacity	Drug Resistance	Metastasis Initiating Capacity
**CD44**	Prostate cancer (100)	Prostate cancer (100) Ovarian cancer (92)	Prostate cancer (100) Ovarian cancer (92)
**CD133+**	Pancreatic cancer (101)	Colorectal cancer (102)	Pancreatic cancer (101)
**ABCG2**	Colon cancer (103)	Colon cancer (103) Esophageal squamous cancer cells (104)	Esophageal squamous cancer cells (104)
**CD49f**	Triple negative breast cancer (105)	Triple negative breast cancer (105)	Human cervical cancer (106)
**CD66**			Human cervical cancer (106) (107),
**OCT4**	Gastric cancer (108) Breast cancer (76)	Gastric cancer (108) Breast cancer (76)	Gastric cancer (108)
**SOX2**	Gastric cancer (108)	Gastric cancer (108)	Gastric cancer (108)
**NANOG**	Breast cancer (76)	Breast cancer (76)	Urinary bladder cancer (109)
**ALDH1A1**	Oral cancer (45)	Ovarian cancer (110)	Breast cancer (78)

Different markers are reported to denote tumor initiating cells, drug-resistant CSCs or metastasis-initiating cells.

In spite of the wide use of the surface molecules like CD133, CD44, ABCG2, CD49f etc. for the identification of CSCs, reporters for these molecules except ABCG2 are not available. For many of these molecules, the stem cell activity is correlated to post-transcriptional regulations (specificity of the isoforms, glycosylation or surface localization). CD133, for example, is a marker for stem cells when it is glycosylated and expressed on cell surface as AC133 epitope (111). In the case of CD44, the splice variants as well as the choice of the ligand are the determinants of cell fate. It has been shown that only hyaluronic acid or osteopontin can initiate signaling pathways downstream of CD44v variant, supporting self-renewal and EMT, while the standard isoform CD44s responds to only hyaluronic acid to induce EMT alone (112). Collectively, many of the surface molecules are not suitable candidates for making reporters depending on their promoter activity. On the other hand, the pluripotency molecules and other molecules like ALDH1A1 impart stemness depending on their level of expression, and thus are ideal candidates for making reporters. So far, there are reports for preclinical studies using reporters for OCT4, SOX2, NANOG, ALDH1A1 and ABCG2, which will be discussed in detail.

## Reporters of CSCs in Preclinical Analysis

### OCT4 Reporter

**Figure 4:**
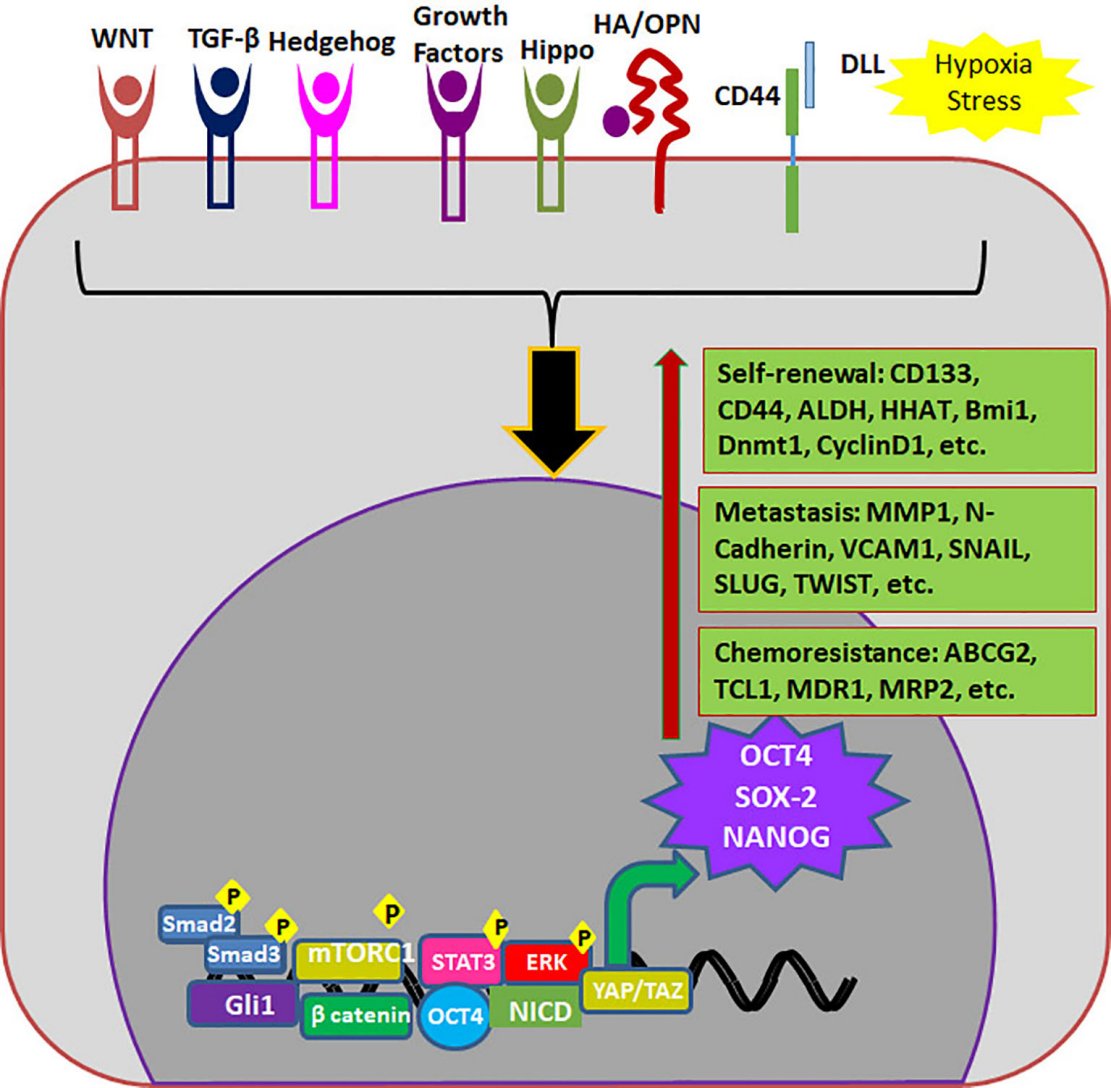
Endogenous markers regulating properties of CSCs. Different signaling pathways regulate the expression of OCT4, SOX2 and NANOG which in turn up-regulates different molecules required for self-renewal, metastasis and chemoresistance.

OCT4 (Octamer binding Transcriptional Factor 4, OCT3, OCT3/4), encoded by *POU5F1* gene is the master regulator of pluripotency in embryonic stem cells. High OCT4 expression is a marker for poor prognosis; aggressiveness, short over-all survival and chemo-resistance, in a variety of malignancies and thus is considered as a CSC marker (**Table 3**) (113). The functional *POU5F1* gene is located on chromosome 6 in humans, while 6 different pseudogenes of it are located at different chromosomes. The expression of OCT4 observed in cancers as mRNA or protein can be misleading since there is high similarity between three OCT4 isoforms and one of the pseudogenes, OCT4-PG1 (113). Different isoforms of OCT4 (OCT4A, B and B1) are coming under the regulation of the same promoter, and are regulated by alternative splicing of exons. While OCT4A regulates self-renewal property, OCT4B and OCT4B1 control stress response in stem cells (114). While some studies report that human *OCT4* promoter is hypermethylated in certain forms of cancer, there are other reports showing that epigenetic mechanisms operate to reactivate the gene in cancer (115, 116). Although the expression of OCT4A is shown in some cancers using immunohistochemistry, a data to support its dimerization with SOX2 in cancer cells to induce self-renewal genes is yet to come (117, 118). At the same time, the over-expression of OCT4B and B1 forms consistent with their anti-proliferative effect, anti-apoptotic function and aggressiveness of the tumor are also reported in various malignancies (117–119). Irrespective of the isoform expressed, OCT4 is considered to be a marker for CSCs and is associated with stemness, chemoresistance and metastatic property of cancer cells (**Table 3**). The different modes of regulation of these properties by OCT4 are summarized in **Figure 4**.

The widely used OCT4 reporter is phOCT4-EGFP generated by Wei Cui, where GFP is under the control of human OCT4 promoter. In human breast cancer, the high expression of this promoter (OCT4^hi^) is shown to mark highly immature cell population possessing self-renewal ability, quiescence, asymmetric division, long doubling time and high metastatic and invasive capacity (61). The same group has shown that this reporter cells can be used to trace dormant breast cancer cells residing in bone marrow, which are responsible for metastasis and tumor recurrence (38). The use of this reporter in tracking metastatic cells was proven in osteosarcoma and colorectal cancer (120, 121). In liver cancer cells, the reporter marks tumor propagating cells showing resistance to Sorafenib (122). Thus this OCT4-EGFP reporter system is useful in real time monitoring drug resistant and metastatic CSCs in animal models. Recently, another reporter system based on OCT4 promoter is reported, where the puromycin expression is driven by OCT4 promoter, and the cells can be selected using puromycin (123). This could be a very useful system to translate to high-throughput platforms for drug screening as the number of cells surviving can be easily measured by different means in automated systems.

### SOX2 Reporter

SOX2 is a transcriptional factor involved in the embryonic development and the generation of iPSCs, which controls the expression of genes required for the maintenance of pluripotency and self-renewal (124). Increased SOX2 expression marks poor prognosis in almost all the cancers, since SOX2 regulates proliferation, EMT, metastasis, tumor initiation, CSC maintenance, resistance to therapy and apoptosis (9). Consistent with that, SOX2 is considered as a CSC marker for a variety of cancers (**Table 2**). The increased expression of SOX2 is attributed more to the gene amplification of chromosome 3q26 than the increased promoter activity (125), except in a few cases like skin squamous cell carcinoma (SCC) (126). The protein product of *PRKCI*, which is amplified along with *SOX2* in the same chromosome location, is shown to activate SOX2 that leads to the activation of target genes like *HHAT* (127) or *PTEN* (128). The major role of SOX2 in the regulation of CSCs is reviewed recently (9) and summarized in **Figure 4**.

Since the over-expression of SOX2 in skin SCC is regulated transcriptionally, a GFP knock-in at the SOX2 chromosomal location can be used to study the role of SOX2 expressing invasive SCC in a mouse model. Using this SOX2–GFP knock-in mouse, it was shown that SOX2-expressing cells in invasive skin SCC are CSCs (126). When gene amplification is involved, instead of the knock-in reporter, another system was used that measure the transcriptional activity of SOX2 (SOX2 SRR2 pGreenFire Response Reporter). Here, SOX2 regulatory region 2 (SRR2), a consensus DNA sequence seen on SOX2 target genes is used to drive GFP. Using this reporter, CSCs were identified in breast cancer and anaplastic large cell lymphoma (129–131), and in breast cancer it is shown to mark CSCs with cisplatin resistance (132).

### SORE6–GFP

In 2014, Tang et al. generated a lentiviral based reporter system depending on the transcriptional activity of both OCT4 and SOX2 where they could functionally denote CSCs. They took six tandem repeats of composite OCT4/SOX2 response element, derived from *NANOG* promoter and cloned it upstream to EGFP fluorescent protein (SORE6-GFP) (133). This system is advanced than the individual OCT4 reporter and SOX2 reporter because the transcriptional activity is regulated by both the factors, which will be a more reliable system to mark CSCs. This reporter identifies CSCs with metastatic potential and chemoresistance in breast cancer (133) gastric cancer (44) and prostate cancer (134). The use of SORE6-GFP in screening drugs to target CSCs was employed in sarcoma models, and they identified EC-8042 as an effective drug to abolish CSCs (43). Recently, a modified version of this reporter is introduced where they have added a FLAG sequence along with GFP, which can be membrane localized, helping in magnetic separation of the cells using antibodies to FLAG (135).

### NANOG-GFP

NANOG is a homeo-box binding transcriptional factor essential for maintenance of pluripotency and self-renewal of embryonic stem cells, being one of the downstream targets of OCT3/4. Even though *NANOG* is silenced in normal somatic cells, aberrant expression is reported in a wide variety of cancers, and it’s up-regulation is correlated to poor survival (5). Consistent with its role in self-renewal and cell reprogramming, it is used as a marker for CSCs in different cancers (**Table 2**) (5). The different modes of regulation of CSC properties by NANOG are represented in **Figure 4**. A reporter for CSCs using human *NANOG* promoter-driven GFP was developed to isolate and characterize triple negative breast cancer stem cells (136). They also used the same reporter to identify CSCs in ovarian cancer, which show resistance to cisplatin (137). Followed by that, different groups started using similar NANOG reporters (39, 138). A lentiviral NANOG-GFP reporter that also expresses luciferase was useful in evaluating drugs that targets colorectal cancer CSCs, both *in vitro* and in mouse xenograft models (138).

### ABCG2 Reporter

ABCG2 is a member of the ATP binding cassette (ABC) transporters, which pumps a wide variety of endogenous and exogenous compounds out of cells (139). ABCG2 is considered as a universal marker of stem cells since it confers side population phenotype associated with stem cells. They can function as membrane transporters, ion channels or receptors (139). The high expression of ABCG2 is observed in a wide variety of cancers, and usually is associated with poor prognosis (63). As mentioned before, ABCG2 is considered as a stem cell marker for CSCs (**Table 2**). There are evidences suggesting that the substrates of ABCG2 may include stem cell differentiation factors thereby retaining the stemness of the cells expressing high ABCG2 (140). Promoter demethylation, histone modification and transcriptional up-regulation by different self-renewal pathways play a major role in the increased activity of ABCG2 in cancer cells (63, 141). Another factor important in clinical relevance is the single nucleotide polymorphism of ABCG2, which critically regulates the pharmacokinetics of different drugs (142).

One of the widely used reporter construct for ABCG2 is ABCG2-Luc, which is used to mark ABCG2 expression and CSCs in different cancers including solid tumors and large B-cell lymphoma (143–145). To study the effect of methylation in the transcriptional regulation of ABCG2, luciferase constructs driven by ABCG2 promoter (pGL3-abcg2-Luc) and *in vitro* methylated ABCG2 promoter was used. The results showed that epigenetic silencing of ABCG2 by methylation is reversed by several chemotherapeutic drugs, resulting in the up-regulation of ABCG2 and acquisition of multidrug resistant phenotype (146). Since transcriptional regulation, at least in part, is driven by epigenetic mechanisms, the stable expression of a reporter driven by unmethylated ABCG2 promoter will not be useful for identification of drug resistant CSCs. Hence, a reporter cell line is recently made using CRISPR-Cas9 gene editing coupled with homology-directed repair. They targeted the EGFP coding sequence to the translational start site of ABCG2, generating ABCG2 knock-out and *in situ* tagged ABCG2 reporter cells (42). This fluorescent reporter system allowed the detection of endogenous regulation of ABCG2 expression by different stress responses and offers a method to screen molecules that can inhibit drug resistant CSCs.

### ALDH1A1 Reporter

Aldehyde dehydrogenase (ALDH) is a family of enzymes that oxidize aldehydes to their corresponding carboxylic acids to prevent oxidative stress in cells. Among the different isoforms, the cytoplasmic variants are responsible for the retinoic acid (RA) biosynthesis, a critical molecule involved in retinoic acid receptor (RAR) signaling, and regulating stemness. RA signaling not only up-regulates cyclin-D1 and c-myc to control cell proliferation, but also protects CSCs from ROS generated under oxidative stress caused by hypoxia. When chemotherapy or radiotherapy is given, the number of ALDH^hi^ cells increases that helps in the oxidization of drug molecules to change its molecular structure. Clinically, ALDH^hi^ expression corresponds to poor prognosis and malignancy in a variety of cancers (147).

**Figure 5:**
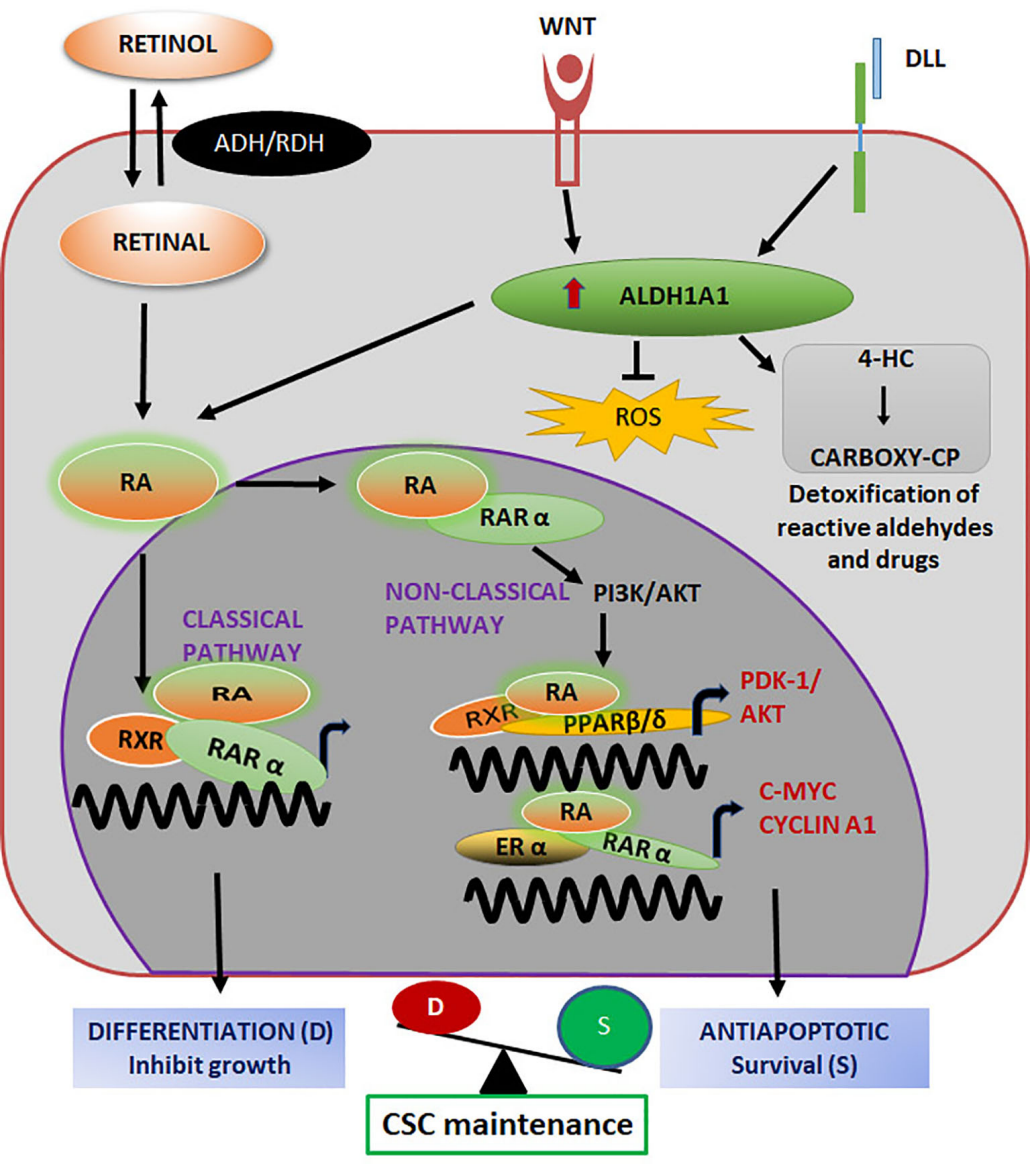
ALDH1A1 in the regulation of CSC properties Both classical and non-classical pathways initiated by Retinol regulate stemness. Retinoic acid (RA) binds to its nuclear receptor RARα and activates their target genes for differentiation. When RA binds to PPARβ/δ/RXR or RARα/ERα, genes related to survival and stemness are up-regulated. The balance of these pathways maintain CSC self-renewal and differentiation. The enzyme activity of ALDH1A1, results in the detoxification of chemotherapeutic drugs to impart chemoresistance.

Retinol is oxidized by all the cytosolic ALDHs to retinaldehyde, while its irreversible conversion to RA is catalyzed by specific ALDH isozymes ALDH1A1, ALDH1A2, ALDH1A3, or ALDH8A1 (148–150). Consistent with that, these isoforms are associated with stemness in various contexts. ALDH1A1 was first identified as a stemness marker in hematopoietic stem cells (151). Later, when the hypothesis of CSCs emerged, it was recognized as a CSC marker in different cancers (**Table 4**). While ALDH1A2 is shown to regulate stem cell properties in neuroblastoma (159), ALDH1A3 over-expression is observed in glioblastoma stem cells (158). Although the specific isotype of ALDH regulating RA signaling in CSCs might vary according to the type of cancer, ALDH1A1 is reported in a wide variety of cancers (**Tables 3**, **4**). ALDH1A1 comes under the regulation of different oncogenic signaling including TGF-β, Notch and WNT pathways and feedback activation by RA signaling (147). Apart from the RAR signaling, ALDH1A1 promotes self-renewing population through tumor growth, self-protection by anti-oxidant activity and development of drug resistance by its catalytic potential (**Figure 5** **)** (147).

**Table 4:** ALDH isoforms as markers of CSCs.

ALDH isoform	Cancer	References
ALDH1A1	Esophageal squamous cell carcinoma	(152)
	Breast cancer	(40)
	Oral cancer	(45)
	Non-small cell lung cancer	(153)
	Ovarian cancer	(110)
	Metastatic melanoma	(154)
	Colon cancer	(40)
ALDH3A1	Melanoma	(155)
	Non-small-cell lung carcinoma (NSCLC)	(155)
	Pancreatic cancer	(156)
ALDH1A3	Breast cancer	(157)
	Glioblastoma	(158)
ALDH1A2	Neuroblastoma	(159)

Different isoforms are reported to denote CSCs in different cancers.

Since majority of the solid tumors express ALDH1A1 in the CSC population, reporter constructs for CSCs based on ALDH1A1 promoter have been reported (40, 45). A reporter construct where tdTomato is driven by ALDH1A1 promoter was used to evaluate drug sensitivity in breast cancer and colon cancer cell lines (40). Further they applied nanotechnology to this reporter system to generate a CSC model to evaluate drug efficiency to target CSCs as a co-culture system with polymer micelles loaded with chemotherapeutic drugs (40). Recently we reported a construct, ALDH1A1-DsRed2, marking CSCs in oral cancer cells to screen inhibitors for their efficacy in targeting CSCs (45). The biochemical analysis coupled with the cytotoxicity evaluation revealed the existence of a signaling cross-talk of several pathways that counteract the effect of one particular pathway inhibitor (45). Thus, it is evident that the outcome of pathway inhibitors predicted by RNAi and over-expression systems may not translate well in therapeutic targeting, suggesting the need to develop *in vitro* an *in vivo* drug screening platforms to target CSCs prior to clinical evaluation.

## CSC Heterogeneity and the Relevance of Reporters

Similar to the hierarchical clustering of stem cells, progenitors and differentiated cells observed during development, distinct states CSCs with different stages of self-renewal and differentiation are predicted for cancer also. There are functionally heterogenic CSCs exhibiting quiescence or dormancy as well as CSCs with a proliferative capacity (66, 160–162). This heterogeneous is considered to be resulting from the CSC plasticity, which is the result of a reprograming initiated by stemness signals in “CSC niche”. The presence of dormant CSCs was noted in several cancers and they are responsible for the relapse of the disease as these cells evade therapy and cause metastasis (163). These dormant CSCs responsible for metastasis in breast cancer can be marked with OCT4GFP reporter (38, 61). At the same time, the CSCs marked with ALDH1A1 are more proliferative in nature (164). ABCG2 is also implicated in the proliferation of stem cells (63). These proliferative CSCs also show multidrug resistance since they express high ALDH and drug efflux molecules. Additionally, CSCs with the hybrid EMT phenotype, marking MICs, express high ALDH (65). In a therapeutic approach, the dormant CSCs and the proliferative, drug resistant, and metastatic CSCs are critical because all these heterogeneous populations can lead to metastasis and recurrence. Considering all these factors, we advocate the use of two reporters, marking OCT4 and ALDH1A1, to identify all the CSCs populations.

## Perspective

For a high-throughput screening, we need to have reporters compatible with automated systems. Fluorescent and luciferase reporters are useful in quantifying the expression level in high-throughput platforms. But when analysis of the expansion or reduction of CSCs as a subpopulation is required, fluorescent reporters may be preferred over luciferase reporters. When we need to take this to preclinical animal models, luciferase reporters and fluorescent reporters of near infrared region, compatible with small animal imaging system, are extremely useful in tracing dormant drug resistant CSCs and metastatic CSCs.

As we discussed in the introduction, the success of a drug in the preclinical screening depends on the screening assay we use. A screening based on the dual reporter will ensure that we consider all the heterogenic CSC populations relevant for recurrence. Using this simple and high-throughput adaptable strategy, we can screen a large number of molecules for CSC-targeting efficiency. The most efficient drugs we obtain after screening has to go through the classical *in vivo* self-renewal assays, because some of the non-CSCs escaping the drug may become CSCs with the influence of a “CSC niche” persisting *in vivo*. The effect of this plasticity has to be taken in to account in drug screening. The introduction of dual reporters for drug screening can tremendously help to increase the number of molecules that enter the drug trial, so that the chance of getting a successful CSC-targeting drug is enhanced.

## Author Contributions

AM collected the data and drafted the manuscript. RR, GM, and PP contributed to the tabulation and graphical representation of the data. TT conceived the idea and critically revised the manuscript. All authors contributed to the article and approved the submitted version.

## Funding

AM received financial support from University Grant Commission, India (19/06/2016 (1) EU-V/318501) and RR received funding from Council of Scientific & Industrial Research, India (09/716(0188)/2019-EMR-I). This work was supported by Department of Science and Technology, India (EMR/2015/001170).

## Conflict of Interest

The authors declare that the research was conducted in the absence of any commercial or financial relationships that could be construed as a potential conflict of interest.
